# The effects of *Crocus sativus* L. (Saffron) and its ingredients on dietary intakes in cardiovascular disease in Iranian population: A systematic review and meta-analysis

**DOI:** 10.3389/fnut.2022.890532

**Published:** 2022-08-04

**Authors:** Majid Kianmehr, Fateme Mahdizadeh, Mohammad Reza Khazdair

**Affiliations:** ^1^Esfarayen Faculty of Medical Sciences, Esfarayen, Iran; ^2^Cardiovascular Diseases Research Center, Birjand University of Medical Sciences, Birjand, Iran

**Keywords:** saffron, crocin, lipid profile, energy, carbohydrate, protein, meta-analysis

## Abstract

Cardiovascular disease (CVD) is one of the most common causes of mortality around the world. The aim of this study is to summarize and conclude the clinical evidence regarding the use of *Crocus sativus* (*C. sativus*) and its ingredients on cardiovascular risk factors. A systematic search was conducted with PubMed, Web of Science (ISI), and Scopus in the English language from 2015 until September 2021. A fixed-effect or random-effects model were applied to pool standard mean difference (SMD) and its 95 % confidence intervals (CI). Randomized controlled studies that assessed the clinical effects of *C. sativus* and its ingredients on dietary intake (Energy, Carbohydrate, Protein, and total Fat) in human subjects were included. Seven studies comprising 421 participants were included in the meta-analysis. Pooling of results showed significant effect of saffron on total fat (−0.14; 95% CI: −0.49 to 0.20; I^2^ = 57.3%) and significant effect of crocin on Energy (0.94; 95% CI: −0.77 to 2.65; I^2^ = 95.9%), Carbohydrate (0.44; 95% CI: −0.74 to 1.62; I^2^ = 92.6%), and Protein (−0.04; 95% CI: −0.26 to 0.34; I^2^ = 0.0%). Present meta-analysis suggests that treatment with crocins is more effective than saffron in energy, carbohydrate, and protein, while saffron is more effective than crocins in fat. However, further studies are needed to confirm these findings.

## Introduction

Cardiovascular disease (CVD) is the most important cause of death in Iran, and indeed most countries scross the world (1). The prevalence of CVD has increased significantly worldwide due to its close association with lifestyle factors such as inactivity, smoking, and poor eating habits (1–3). It is also developing in Iran (4, 5), where in particular abdominal obesity can increase metabolic heart risks through blood pressure, type 2 diabetes, hyperlipidemia, and insulin resistance (6, 7). Control of CVD risk factors reduces mortality from ischemic or coronary heart disease (CHD) because high cholesterol, high blood pressure, smoking, and physical inactivity (of at least 75%) cause new cases of CHD. These findings are encouraging individuals to lead a healthy lifestyle. In other words, healthy eating habits and tobacco, including smoking, have been characterized by the WHO as one of the biggest public health threats (8). In addition, CVD reduces patient life expectancy as well as induces a financial burden on patients and governments (9). Therefore, it is essential for any health care system to adopt appropriate strategies for the management and treatment of CVD and related diseases (10).

*Crocus sativus* L (*C. sativus*), known as saffron, is a small perennial herb belonging to the family *Iridacea*. This plant is cultivated in many countries with mild and dry climates, especially Iran, Afghanistan, Turkey, Greece, and Spain (11, 12). This herb is native to Iran (13) was used traditionally as a medicine beginning more than 3,600 years ago (14).

According to chemical analysis, there are more than 150 components in *C. sativus* stigma, including crocins and crocetin (carotenoids). Crocins are members of a family of molecules which are formed from crocetin which is responsible for the yellow color of saffron (15). Picrocrocin (apocarotenoid) which gives saffron its flavor, and safranal (monoterpene Aldehyde) which is responsible for the aroma, were also identified (16–18).

Various medicinal properties of saffron and its compounds have been extensively studied, including anti-cancer (19, 20), anti-depressant, anti-Parkinson's, anti-ischemic (such as brain, kidney (21), muscle, and heart ischemia), anti-hypertensive, anti-genotoxic and antidote (eg against snake venom, diazinon, acrylamide, or acrolein), antitussive, hypolipidemic, anti-inflammatory (18, 22, 23), anti-Alzheimer's (24), and antioxidant (25, 26). Also, the protective effects of saffron and its components on the respiratory system have been reported (12, 27).

In traditional medicine in different countries, saffron is used for various purposes such as analgesic and anti-inflammatory (earache, toothache, swelling, otitis, anal pain, gout, cancer pain, gingivitis), cardiovascular system, eye disease (painful eye, day blindness, corneal disease and cataract, eye infections, and poor vision), gastrointestinal system (stomachic, anorexia, treatment of hemorrhoid, jaundice, anti-flatulence, and enlargement of liver), genitourinary system (abortion, treatment of amenorrhea, use in the puerperium period, terminate pregnancy, painful urination, diuretics, and kidney stones), infectious disease (antibacterial, antiseptic, antifungal, measles, smallpox, and scarlet fever), respiratory system (Asthma, bronchitis, expectorant, pertussis, and antitussive), skin Disease (treatment of psoriasis, eczema, and acne) (28), and other cases (Immunostimulant and tissue coloration) (18), relieving symptoms of PMS (29), and central nervous system (anti-hysterical, CNS stimulant, hypnotic, mental disease, sedative and anticonvulsant (30, 31).

As mentioned earlier, CVD is the leading cause of death worldwide (1). Platelet activation and accumulation play a key role in homeostasis and thrombosis. Herbal remedies have long been used to treat a variety of ailments, including cardiovascular disease (32). Herbs can play an important role in improving the progression of cardiovascular disease, especially platelet function, as well as some coagulation parameters. Herbal remedies such as feverfew, garlic, ginger, ginseng, and willow bark have been found to be effective in cardiovascular diseases (33). Saffron has been traditionally used to improve cardiovascular functions, increase heart health, and in treatment of palpitation (34). In the current study, we conducted a meta-analysis to determine the effects of saffron and its derivatives on cardiovascular disease. The findings of this study can help us find new herbal remedies for cardiovascular disorders. The efficacy of saffron and its main carotenoid, crocin on lipid profile, appetite level, dietary intakes, anthropometric indices, and body composition in patients with coronary artery disease were evaluated.

## Methods

### Search strategy

The present study was performed according to the Preferred Reporting Items for Systematic reviews and Meta-Analyses (PRISMA), (35, 36).

We searched the published studies in databases such as; PubMed, Web of Science (ISI), and Scopus in the English language until September 2021.

TITLE-ABS-KEY “crocin” OR TITLE-ABS-KEY “crocetin” OR TITLE-ABS-KEY “saffron” OR TITLE-ABS-KEY “*Crocus sativus* linn” AND TITLE-ABS-KEY “hypertension” OR TITLE-ABS-KEY “ blood pressure” in the Scopus:105

“crocin” [Title/Abstract] OR “crocetin” [Title/Abstract] OR “saffron” [Title/Abstract] OR “*Crocus sativus* linn” [Title/Abstract] AND “hypertension” [MeSH Terms] OR “blood pressure” [Title/Abstract] in PubMed: 75

TS= “crocin” OR “crocetin” OR “saffron” OR “*Crocus sativus* linn” AND TS= “hypertension” OR “blood pressure” in ISI=85.

### Inclusion criteria

All clinical studies that investigated the effect of crocin, crocetin (or saffron, *Crocus sativus* linn) on coronary artery disease (CAD) patients were included in the study.

### Exclusion criteria

Review articles, editorials, duplicates, animal studies, and clinical studies about the effect of crocin (or saffron, *Crocus sativus* linn) on coronary artery disease (CAD) patients without a control group were excluded from the study.

### Statistical analysis

The heterogeneity between studies in a meta-analysis will exist. This heterogeneity may be of clinical, methodological, or statistical origin. Quantifying statistical heterogeneity through I^2^-statistics is only helpful when the amount of clinical heterogeneity is unknown and I^2^ is high (37). Given statistical heterogeneity between studies, fixed-effect, or random-effects models were done, and calculate the standardized mean differences (SMD) and its 95% confidence intervals (CI), (38). To assess the statistical heterogeneity, I^2^ > 50% (high) and *P*-value <0.05 (significant) were used (39).

The SMD is used when reporting effectiveness studies in terms of continuous measurement. The SMD is known as Cohen's d (40). The SMD is computed with this formula (41):

SMD = (new treatment improvement – comparator (placebo) improvement)/pooled standard deviation (41). A *P*-value <0.05 is considered statistically significant.

## Results

### Literature search

**Figure 1:**
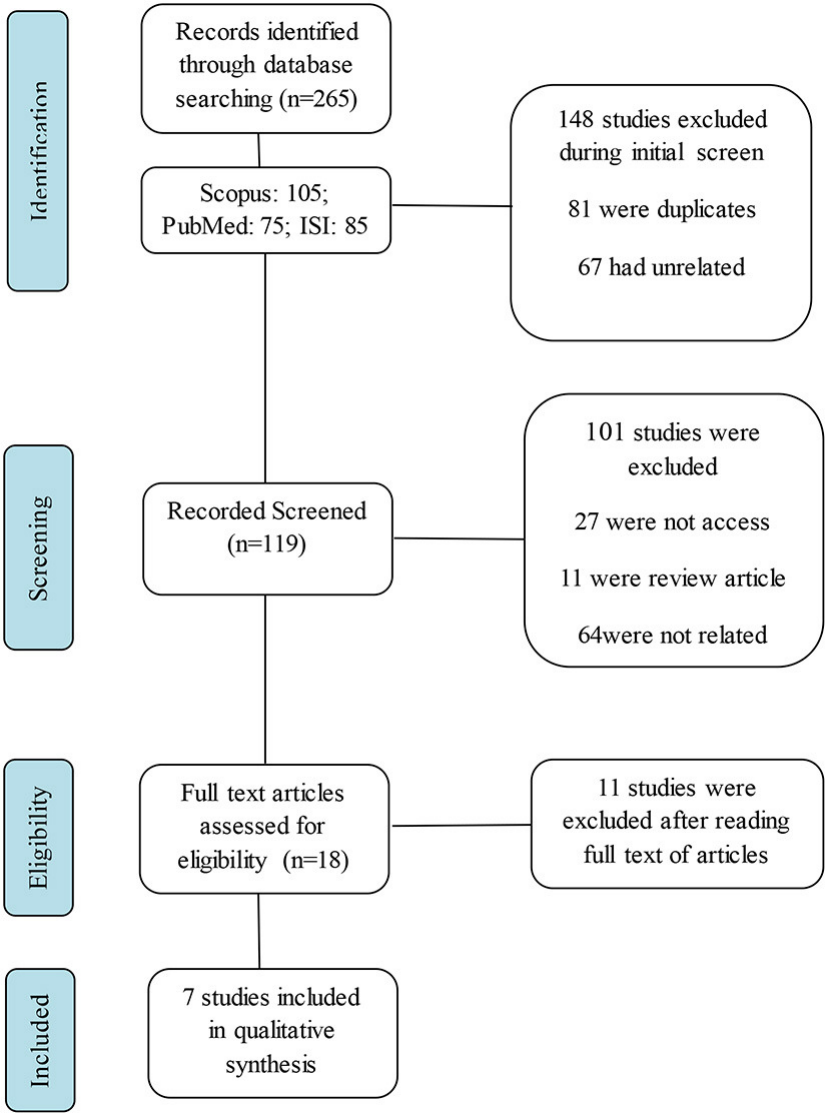
Flowchart of the literature search and strategy for the selection of relevant studies.

Initially, 265 articles were extracted. All unrelated studies including; duplicates, animal, and review studies as well as clinical studies without a control group were removed and seven articles were considered for further evaluation after reviewing the titles and abstracts of the extracted articles (12, 41, −47), the process of study selection is shown in Figure 1.

### Trial characteristics

Demographic characteristics of the seven included trials containing 412 subjects that were published between 2016 and 2021 are shown in Table 1. The mean age of participants ranged between 52.02 to 56.32 years old. The treatment period was 8 or 12 weeks (13, 42–48). The Nutritionist IV for Windows software was used to analyze energy and macronutrient compositions (The Hearst Corporation, San Bruno, California.) in the trials (44).

**Table 1:** Demographic characteristics of the included studies.

	**Author/year**	**Country**	**Mean age (years)**	**Gender**	**Sample size**	**Duration**	**Randomization**
1	Milajerdi et al. (42)	Iran	54.57 ± 6.96	Male and female	27	8 weeks	Double-blind, randomized, and placebo-controlled clinical trial
			54.42 ± 7.58		27		
2	Azimi et al. (43)	Iran	52.02 ± 1.0	Male and female	42	8 weeks	Single-blind, randomized, placebo-controlled, clinical trial
			53.64 ± 1.3		39		
3	Abedimanesh et al. (44)	Iran	56.04 ± 7.55	Male and female	25	8 weeks	Randomized, double-blind, placebo-controlled trial
			56.32 ± 5.91		25		
4	Milajerdi et al. (45)	Iran	54.57 ± 6.96	Male and female	26	8 weeks	Triple Blind, randomized clinical trial
			54.42 ± 7.58		26		
5	Ebrahimi et al. (46)	Iran	55.2 ± 7.3	Male and female	40	12 weeks	Double-Blind, randomized clinical trial
			53 ± 10.6		40		
6	Abedimanesh et al. (47)	Iran	54.83 ± 5.99	Male and female	24	8 weeks	A pilot, randomized, double-blind, placebo-controlled clinical trial
			56.00 ± 5.67		21		
7	Behrouz et al. (48)	Iran	57.08 ± 7.41	Male and female	25	12 weeks	A pilot study, randomized, double-blind, parallel-group, clinical trial
			59.86 ± 9.46		25		

### Meta-analysis

#### The association between saffron and energy

**Figure 2:**
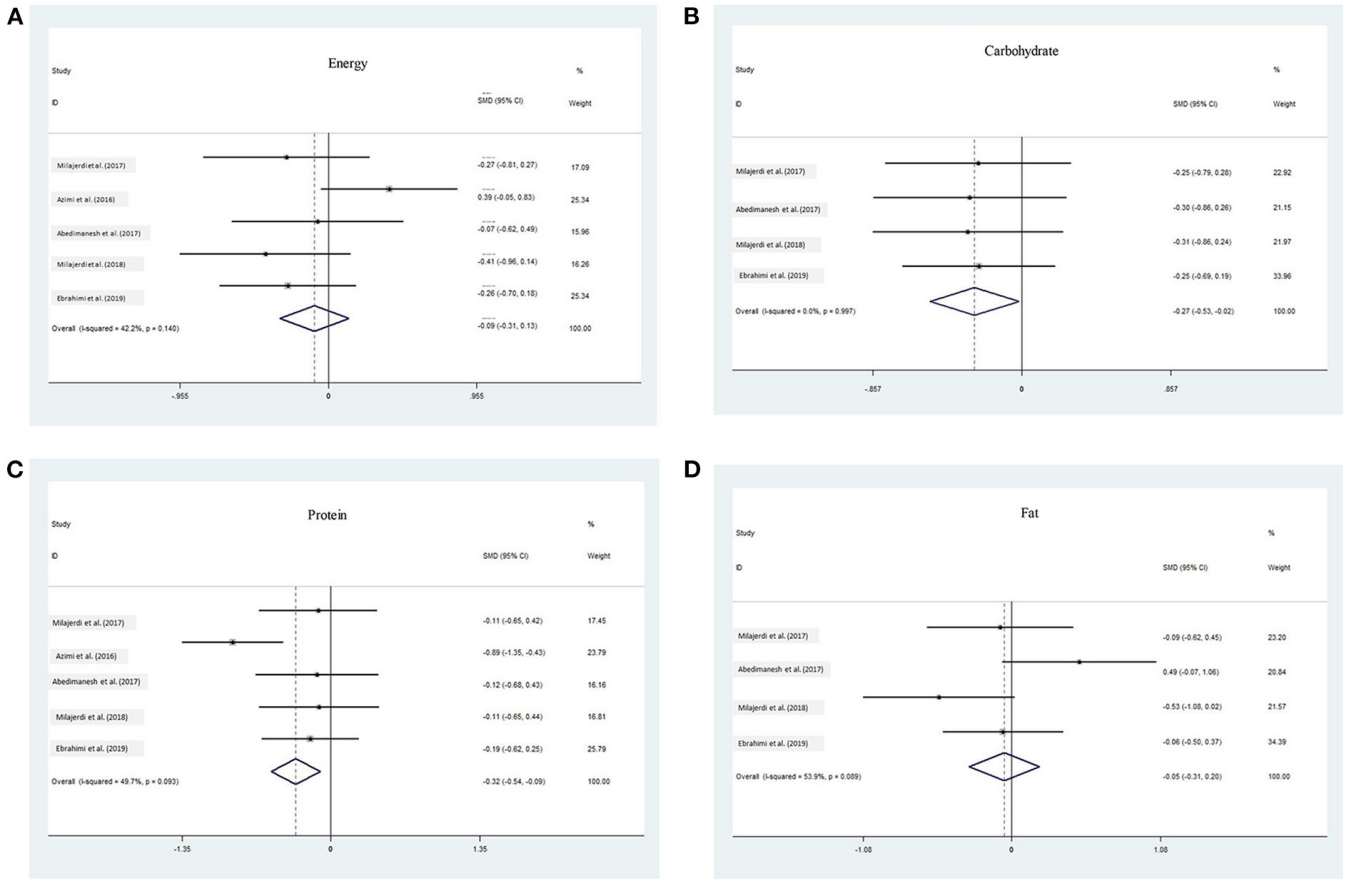
The association between saffron and **(A)** Energy, **(B)** carbohydrate, **(C)** protein, and **(D)** fat.

Combining the results of 5 analytic studies using a fix effect model (I^2^ = 42.2%, Q = 6.92, *P* = 0.140), showed the pooled SMD for energy were 0.09 fold lower in saffron group (95% confidence interval:-0.310, 0.133) (Figure 2A). Based on the Egger regression plot (*t* =-1.05, *p* = 0.373) no publication bias existed.

#### The association between saffron and carbohydrate

Fix effect meta-analysis of four studies (I^2^ = 0.0%, Q = 0.05, *P* = 0.997) showed that carbohydrate were 0.27 fold lower in saffron than control group (pooled SMD: −0.27; 95% CI:−0.53,-0.02) (Figure 2B). Based on the egger test (*t* =-1.56, *p* = 0.259), no publication bias existed.

#### The association between saffron and protein

Combining the results of five analytic studies using a fix effect model (I^2^ = 49.7%, Q= 7.95, *P* = 0.093), showed the pooled SMD for energy were 0.32 fold lower in saffron group than control group (95% CI: −0.54, −0.09) (Figure 2C). Based on the Egger regression plot (t= 1.07, p = 0.364) no publication bias existed.

#### The association between saffron and total fat

Fix effect meta-analysis of four studies (I^2^ = 53.9%, Q = 6.51, *P* = 0.09) showed that fat were 0.06 fold lower in saffron group (95% CI: −0.31, 0.20) (Figure 2D). The Egger test showed (*t* = 0.13, *p* = 0.908), no publication bias existed (Table 2).

**Table 2:** Summary of effect estimates of *C. sativus* (saffron) for dietary intakes.

**Author/date**	**Dosage**	**Patients/numbers**	**Intervention**	**Duration**	**Results**
Milajerdi et al. (42)	15 mg/day	54	Hydro-alcoholic extract	8 weeks	Uric acid and blood urea nitrogen were significantly decreased in the saffron-treated group. Blood pressure, dietary intake, and physical activity had no significant changes observed between saffron and placebo-treated groups. Moreover, AST, ALT, and ALP between the two groups were non-significantly changed.
Azimi et al. (43)	1 gram	81 people with T2DM	Saffron	8 weeks	There were no significant differences seen in mean physical activity (MET-h/wk), energy intakes, or macro- and micronutrients between saffron and control groups. Treatment with saffron significant effect on intercellular adhesion molecule-1(ICAM-1) concentrations (340.9 ± 14.4 ng/ml vs. 339.69 ± 14.4 ng/ml).
Abedimanesh et al. (44)	30 mg	50 patients with CAD	Saffron aqueous extract (SAE)	8 weeks	Saffron improved anthropometric and body composition variables. Weight loss was significantly higher in the SAE group. Moreover, SAE decreased BMI, energy, dietary intake, mean values, and the feeling of hunger, waist circumference, fat mass, and fat-free mass mean values significantly compared to the placebo group. Also, the feeling of fullness and satiety in the SAE group increased dramatically compared to placebo-treated group.
Milajerdi et al. (45)	15 mg	54T2D patients	Saffron hydroalcoholic extracts	8 weeks	The physical activity and dietary intakes (Energy, total carbohydrate, total protein, and total fat) were no significant differences between the saffron and placebo groups. Moreover, the extract of saffron reduces the serum concentration of FBS as compared to the placebo group.
Ebrahimi et al. (46)	100 mg/day	80 T2D patients	Saffron	12 weeks	Saffron supplementation significantly reduced systolic blood pressure (SBP). Liver enzymes, serum creatinine, serum urea, 24-h urine albumin, diastolic blood pressure (DBP), and physical activity were not significantly different between the saffron and placebo groups.However, the saffron extract reduced dietary intake, but these changes were not significant.

#### The association between saffron ingredients on energy

**Figure 3:**
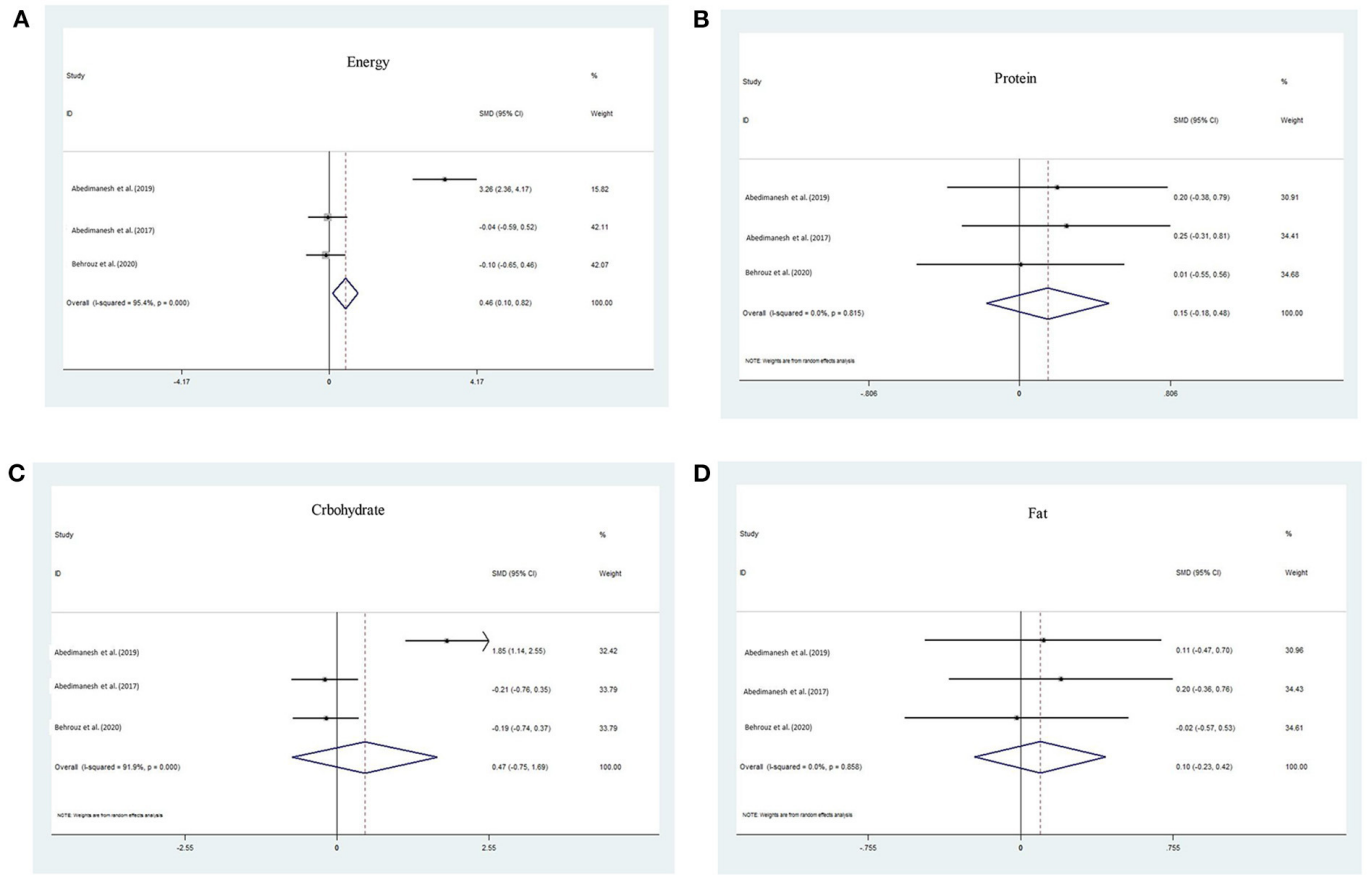
The association between crocin and crocetin and **(A)** Energy, **(B)** carbohydrate, **(C)** protein, and **(D)** fat.

Combining the results of three analytic studies using a fix effect model (I^2^ = 95.4%, Q = 43.83, P = 0.000), showed the pooled SMD for energy was increased 0.459 fold in the crocin group over the control group (95% CI: 0.1, 0.819) (Figure 3A). Based on the Egger regression plot (*t* = 44.32, *p* = 0.02).

#### The association between saffron ingredients on carbohydrate

Random effect meta-analysis of three studies (I2 = 91.9%, Q= 24.69, *P* = 0.00) showed that carbohydrate were 0.47 fold increase in crocin than control group (pooled SMD: 0.47; 95% CI: −0.75, 1.69) (Figure 3B). Based on the egger test (*t* = 96.11, *p* = 0.007).

#### The association between saffron ingredients on protein

Random effect meta-analysis of three studies (I^2^ = 0.0%, Q = 0.41, *P* = 0.815) showed that protein were 0.15 fold increase in crocin than control group (95% CI: −0.18, 0.48) (Figure 3C). Based on the eggertest (*t* = 0.42, *p* = 0.748), no publication bias existed.

#### The association between saffron ingredients on total fat

Random effect meta-analysis of three studies (I^2^ = 0.0%, Q = 0.31, *P* = 0.858) showed that fat were 0.01 fold increase in crocin than control group (95% CI: −0.23, 0.42) (Figure 3D). Based on the egger test (*t* = 0.16, *p* = 0.9), no publication bias existed (Table 3).

**Table 3:** Summary of effect estimates of saffron ingredients (crocin and crocetin) for dietary intakes.

**Author/date**	**Dosage**	**Patients/numbers**	**Intervention**	**Duration**	**Results**
Abedimanesh et al. (47)	10 mg/day	45 CAD patients	Capsule of Crocetin	8 weeks	The mean values of total energy, carbohydrate, total fat, and protein intake were significantly decreased in the crocetin group. Also, all the feelings of hunger and fullness were decreased while the feeling of satiety significantly increased in the crocetin group compared to the placebo group after adjusting for age and sex. Serum circulating homocysteine (Hcy), heart-type fatty-acid-binding protein (h-FABP), and systolic and diastolic blood pressures decreased significantly in the crocetin group, while high-density lipoprotein (HDL) significantly increased compared to the placebo group.
Abedimanesh et al. (44)	30 mg	50 CAD patients	Crocin capsules	8 weeks	Crocin improved the anthropometric and body composition variables compared to the placebo group. Crocin significantly decreased BMI, waist circumference, fat mass, fat-free mass mean values, energy, dietary intake mean values, and the feeling of hunger significantly compared to the placebo group. Also, feelings of fullness and satiety in the crocin group increased dramatically compared to placebo-treated group.
Behrouz et al. (48)	15 mg twice daily (30 mg/day)	50 T2D patients	Crocin tablets	12 weeks	Crocin supplementation reduced dietary intakes (Energy, Total Carbohydrate, Total Protein, and Total Fat) compared to the control group but these changes were not significant. Also, crocin led to significant improvement in plasma levels of glucose, insulin, hemoglobin A1c, systolic blood pressure, insulin resistance, and insulin sensitivity.

## Discussion

To the best of our knowledge, the present systematic review and meta-analysis is the first study that evaluated the association between saffron and its ingredients with dietary intakes (energy, carbohydrate, protein, and total fat).

The meta-analysis of data from five clinical trials of saffron showed that the SMD of energy, carbohydrate, and protein was lower in saffron groups than in the control which means that treatment is more effective than a placebo, but for fat, the treatment is less effective than a placebo.

The meta-analysis of data from three clinical trials of crocins showed that the SMD of energy, carbohydrate, protein, and fat was increased in crocin groups than compared to controls, which means that treatment is more efficacious than a placebo, but for fat the treatment is less efficacious than a placebo. The study by Abedimanesh et al. has found that saffron aqueous extract (SAE) and crocin significantly decreased energy and dietary intake mean values in patients with CAD in comparison to a placebo-treated group. In addition, the feeling of hunger decreased significantly in SAE and crocin groups. These changes in the SAE treated group were more efficacious than the crocin group in energy, carbohydrate, and protein intake (44). Some other active components (crocetin and safranal) in addition to crocin may be involved in the effects of the saffron extract on these properties. It may be due to synergistic interactions between bioactive molecules within the saffron composition. The results of the other study also showed that supplementation with saffron powder reduced energy intake, carbohydrate, protein, and total fat in patients with type 2 diabetes (T2D), but these changes were not significant (46). Differences in the duration of the studies and type of diseases may be reasons for these different results.

Saffron ethanolic extract and crocin in a rat model of high-fat, diet-induced obesity restricted appetite and food consumption (49). The results of one study suggested that both saffron and crocin reduced the body weight, food intake, and blood leptin levels significantly compared to the control group and baseline in adult male Wistar rats, and also the effects were comparable to sibutramine (is a serotonin and norepinephrine reuptake inhibitor which has been used for short- and long-term therapy of obesity) (50). These results confirmed the anorexigenic effect of saffron and its active ingredients.

Obesity is a significant risk factor for CVD (51). The relationship between macronutrients and cardiovascular disease and mortality in a Prospective Urban Rural Epidemiology study showed that high carbohydrate intake was associated with a higher risk of total mortality, whereas total fat and individual types of fat were related to lower total mortality (52). It has been reported that a high carbohydrate, low protein diet enhanced cardiovascular disease risk compared to a moderate carbohydrate, moderate protein weight loss diet in obese adults (53). It has been reported that high-carbohydrate or high-fat diets have more deleterious metabolic effects. Large randomized controlled trials have shown that a reduction of fat intake as part of a healthy lifestyle combined with weight reduction reduces the risk of diabetes and cardiovascular disease (54).

It has been reported that active components in saffron, such as crocin solely or synergic with other ingredients (picrocrocin, safranal, etc.) can reduce food intake and blood leptin levels as well as modulate neurotransmitter pathways, especially serotonin reuptake (50) and probably it can target appetite control centers and limit food intake.

The results of the current meta-analysis indicate that saffron and its ingredients, due to reduction in energy and dietary intake mean values, restriction of appetite and food consumption in patients with CAD, could reduce the risk factor for CAD (34).

Many studies have examined the properties between crocin or saffron and CVD. Hatziagapiou and Lambrou indicated the role of *C. sativus* and/or its active constituents (in particular, crocin) as a cardiovascular-protective agent, and this study shows the beneficial results of saffron consumption against ischemia-reperfusion injury (IRI) hyperlipidemia and diabetes due to up-regulation of antioxidant parameters (superoxide dismutase and catalase activity) and anti-inflammatory mediators (IL-10) as well as reduced pro-inflammatory mediators (TNF-α, IL-1β, and IL-6) and anti-apoptotic markers (55). In addition, SAE and crocin could improve the quality of life and depression in patients with CAD (56, 57). These activities might be a reason for body weight loss as a consequence of saffron supplementation. The results of *in-vitro* study suggested several possible mechanisms for saffron cardio protective properties. Saffron or its main constituents, might reduce blood lipids *via* inhibiting pancreatic and gastric lipase activity which is a key enzyme for fat absorption and increasing fecal excretion of fat (58). Saffron and its main constituents (crocins, crocetin, and safranal) can alleviate oxidative stress and inflammatory proses, which are associated with the risk of cardiovascular disease. In addition, given that the main pharmacological activity of saffron is attributed to its main constituents.

The results of the current study suggested that crocins are more effective than saffron in dietary intake (energy, carbohydrate, and protein), but because of the safety, easy use, and low price, use of saffron as a spice and food additive suggests instead of its main components such as crocins.

While saffron is an expensive spice, the variety of its biological properties including, anti-oxidation, anti-inflammation, antidepressant, and hypolipidemic actions suggest that the benefits of using saffron outweigh its high price.

## Conclusion

The present evidence suggests that saffron and its active constituents might be beneficial for dietary intake (Energy, Carbohydrate, Protein, and total Fat) and support some aspects underlying cardiovascular protective properties of saffron. It should be noted that this study is the first study investigating the effect of saffron and crocin on dietary intakes among CVD patients, therefore, further studies are needed to confirm these findings.

## Data availability statement

The original contributions presented in the study are included in the article, further inquires can be directed to the corresponding author.

## Author contributions

MK searched the literature and helped in the preparation of the manuscript. FM searched literature and analyzed the results. MRK was responsible for study design, searching the literature, and prepared and revised the manuscript. All authors contributed to the article and approved the submitted version.

## Conflict of interest

The authors declare that the research was conducted in the absence of any commercial or financial relationships that could be construed as a potential conflict of interest.

## Publisher's note

All claims expressed in this article are solely those of the authors and do not necessarily represent those of their affiliated organizations, or those of the publisher, the editors and the reviewers. Any product that may be evaluated in this article, or claim that may be made by its manufacturer, is not guaranteed or endorsed by the publisher.
